# Shot and Patronin polarise microtubules to direct membrane traffic and biogenesis of microvilli in epithelia

**DOI:** 10.1242/jcs.189076

**Published:** 2016-07-01

**Authors:** Ichha Khanal, Ahmed Elbediwy, Maria del Carmen Diaz de la Loza, Georgina C. Fletcher, Barry J. Thompson

**Affiliations:** The Francis Crick Institute, 44 Lincoln's Inn Fields, London WC2A 3LY, UK

**Keywords:** *Drosophila*, Epithelia, Polarity, Microtubules, Microvilli, Spectrin

## Abstract

In epithelial tissues, polarisation of microtubules and actin microvilli occurs along the apical-basal axis of each cell, yet how these cytoskeletal polarisation events are coordinated remains unclear. Here, we examine the hierarchy of events during cytoskeletal polarisation in *Drosophila melanogaster* epithelia. Core apical-basal polarity determinants polarise the spectrin cytoskeleton to recruit the microtubule-binding proteins Patronin (CAMSAP1, CAMSAP2 and CAMPSAP3 in humans) and Shortstop [Shot; MACF1 and BPAG1 (also known as DST) in humans] to the apical membrane domain. Patronin and Shot then act to polarise microtubules along the apical-basal axis to enable apical transport of Rab11 endosomes by the Nuf–Dynein microtubule motor complex. Finally, Rab11 endosomes are transferred to the MyoV (also known as Didum in *Drosophila*) actin motor to deliver the key microvillar determinant Cadherin 99C to the apical membrane to organise the biogenesis of actin microvilli.

## Abstract (precis)

**Summary:** In *Drosophila*, microtubules are polarised along the apical-basal axis of epithelial cells by the concerted action of Shot and Patronin, two apically localised microtubule binding proteins.

## INTRODUCTION

Cells in epithelial tissues are polarised and display distinct apical and basolateral membrane domains (Martin-Belmonte and Mostov, 2008; Rodriguez-Boulan and Macara, 2014; St Johnston and Ahringer, 2010; Tepass, 2012). How this fundamental apical-basal polarity is elaborated to direct the polarisation of all other features of epithelial cells remains a major unsolved problem (Nance and Zallen, 2011). For example, many epithelial cells exhibit polarisation of the spectrin and microtubule cytoskeletons along the apical-basal axis, as well as polarisation of the actin cytoskeleton to produce distinctive apical microvilli. Although the molecular assembly of spectrins, F-actin microvilli and acentrosomal microtubules have been intensely studied, how these cytoskeletal features become polarised remains unclear (Bartolini and Gundersen, 2006; Sauvanet et al., 2015; Suozzi et al., 2012; Thomas, 2001).

In the case of the spectrin cytoskeleton, polarisation was first observed in *Drosophila* epithelial cells, where an apical β-Heavy (β_H_)-Spectrin subunit and basolateral β-Spectrin subunit segregate into complementary cortical domains (Lee et al., 1997; Thomas and Kiehart, 1994). Both types of β-subunit can dimerise with α-Spectrin to form a spring-like network that interacts with FERM domain proteins and transmembrane proteins such as Crumbs (reviewed in Bennett and Healy, 2009). Spectrins have been shown to function in maintaining membrane tension and in regulating signalling through the Crumbs–Hippo pathway, but whether there is a role for spectrins in controlling apical-basal polarity has proven elusive (Deng et al., 2015; Fletcher et al., 2015; Krieg et al., 2014; Médina et al., 2002; Thomas et al., 1998; Wong et al., 2015; Zarnescu and Thomas, 1999). Recent work has suggested that basolateral spectrins act with Integrins to promote columnar cell shape in *Drosophila* follicle cells (Ng et al., 2016). The apical FERM domain proteins have been linked to organisation of the actin cytoskeleton and microvilli in both *Drosophila* and mammalian cells, but whether the spectrin cytoskeleton is also involved in this process remains unclear (Claret et al., 2014; Gloerich et al., 2012; Hipfner et al., 2004; Karagiosis and Ready, 2004; Polesello et al., 2002; Roch et al., 2010; Speck et al., 2003; ten Klooster et al., 2009).

In the case of the microtubule cytoskeleton in epithelial cells, the centrosomal nucleation of the mitotic spindle evident during mitosis gives way to an acentrosomal nucleation of polarised microtubules at the apical and basal plasma membranes during interphase, a process first noticed by electron microscopy studies in *Drosophila* (Mogensen and Tucker, 1987; Mogensen et al., 1993, 1989). Acentrosomal nucleation of microtubules was later demonstrated to occur in many eukaryotic organisms, from yeast to human cells (Carazo-Salas and Nurse, 2006; Mahoney et al., 2006; Reilein et al., 2005; Schuh and Ellenberg, 2007; Stiess et al., 2010). Nevertheless, the molecular system responsible for polarising microtubules in epithelial cells is still to be identified. Consequently, it has been difficult to genetically test the functional role of polarised microtubules in epithelia. Instead, mutation of the microtubule minus-end-directed motor protein Dynein, or its adaptors, has been used to demonstrate a requirement for polarised microtubules in apical mRNA transport and positioning of the nucleus in *Drosophila* epithelia (Bullock and Ish-Horowicz, 2001; Dix et al., 2013; Holt and Bullock, 2009; Horne-Badovinac and Bilder, 2008; Liu et al., 2013; Mosley-Bishop et al., 1999; Swan et al., 1999; Wilkie and Davis, 2001). There also appears to be a role for Dynein in trafficking E-cadherin during early polarity establishment and during tracheal morphogenesis (Harris and Peifer, 2005; Le Droguen et al., 2015). The overall apical-basal polarisation of epithelial cells is sometimes affected in *dynein* mutants, which resemble *crumbs* mutants, which mostly polarise normally but occasionally lose polarity and become multilayered (Bullock and Ish-Horowicz, 2001; Fletcher et al., 2012; Horne-Badovinac and Bilder, 2008; Wilkie and Davis, 2001). Accordingly it has been proposed that Dynein traffics mRNA encoding Stardust, a Crumbs-binding partner (Horne-Badovinac and Bilder, 2008). These results raise the question of whether polarised microtubules are truly essential for polarised trafficking and localisation of membrane proteins, as has often been suggested based on observations of membrane trafficking in mammalian epithelial cells in culture (Mostov et al., 2000; Rodriguez-Boulan et al., 2005).

In the case of apical microvilli, the specific microvillar protocadherin PCDH15 was identified in human genetic studies of Usher syndrome, an inherited deaf-blindness disease caused by defects in stereocilia of the human ear cochlear cells and microvilli of the eye photoreceptor cells (Alagramam et al., 2001a,b; Ben-Yosef et al., 2003). PCDH15 interacts with CDH23 to form tip-link filaments in stereocilia that are necessary for hearing (Elledge et al., 2010; Geng et al., 2013; Kazmierczak et al., 2007; Söllner et al., 2004). The *Drosophila* PCDH15 homologue is named Cadherin 99C (Cad99C) and is necessary for normal biogenesis of microvilli, and is also sufficient to expand microvilli length when overexpressed (Chung and Andrew, 2014; D'Alterio et al., 2005; Schlichting et al., 2006). To perform its function, Cad99C/PCDH15 interacts with the actin motor protein Crinkled/MyosinVIIA, which is encoded by the human *MYO7A* gene that is also mutated in Usher syndrome patients (Glowinski et al., 2014). However, it remains a mystery how Cad99C/PCDH15 becomes localised to the apical domain of epithelial cells.

Here, we show that polarised microtubules are essential to direct trafficking of Cad99C to apical microvilli in *Drosophila*. We identify the microtubule-binding proteins Patronin (CAMSAP1, CAMSAP2 and CAMSAP3 in humans) and Shortstop [Shot; MACF1 and BPAG1 (also known as DST) in humans] as acting in parallel at the apical domain of epithelial cells to polarise microtubules and delivery of Cad99C. We further show that polarisation of Patronin and Shot is dependent on the apical spectrin cytoskeleton, which in turn is dependent of determinants of cell polarity. Cad99C is transported apically inside Rab11 endosomes, which are linked to the Dynein microtubule motor protein through its adaptor protein nuclear fallout (Nuf). Once at the apical cortex, Rab11 endosomes are transferred to the MyoV (known as Didum in *Drosophila*) actin motor complex to enable delivery of Cad99C to the apical plasma membrane. Our findings reveal a new mechanism linking epithelial cell polarity with the polarisation of the spectrin and microtubule cytoskeleton to direct apical membrane trafficking and biogenesis of microvilli.

## RESULTS

**Fig. 1.**
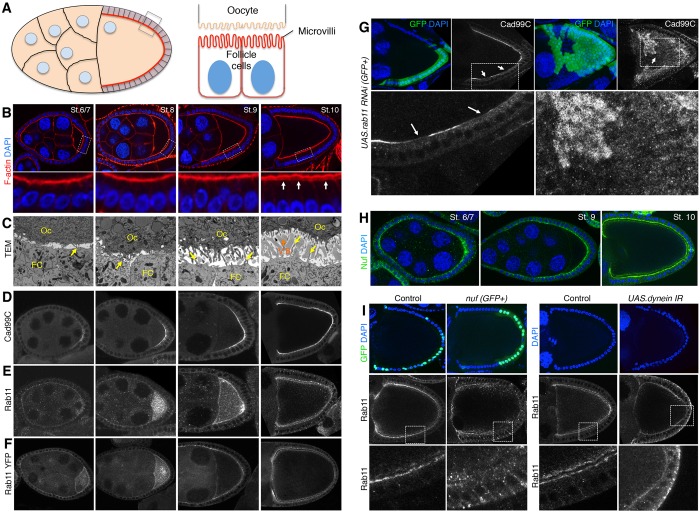
**Cad99C is trafficked to the apical membrane via Rab11 endosomes during microvilli morphogenesis.** (A) Schematic diagram of a stage 10 *Drosophila* egg chamber, highlighting cells that make microvilli. (B) Wild-type egg chambers at different stages of oogenesis stained for DAPI to mark nuclei and F-actin to visualise the apical actin-rich microvilli (arrows). (C) TEM images of wild-type egg chambers at different stages of microvilli biogenesis. Arrows point to apical microvilli in follicle cells. Oc, oocyte; FC, follicle cells; V.B., vitelline bodies. Cad99C (D) and Rab11 (E) become polarised apically during stages of microvilli biogenesis in wild-type egg chambers. (F) Egg chambers expressing a knock-in Rab11–YFP insertion in the endogenous gene. (G) Expression of Rab11 RNAi (GFP-positive clone) causes mislocalisation of Cad99C (arrows). (H) Nuf is localised apically during stages of microvilli biogenesis. (I) Mutation of *nuf* (GFP-positive clone) or knockdown of Dynein (whole egg chamber) causes mislocalisation of Rab11 endosomes.

We begun by examining the biogenesis of apical microvilli in the *Drosophila* ovarian follicle cell epithelium. As previously reported by others, we found that during mid-oogenesis, Cad99C localises specifically to the apical domain of follicle cells that are initiating biogenesis of apical microvilli (D'Alterio et al., 2005; Schlichting et al., 2006). These Cad99C-positive microvilli are visible upon staining for filamentous actin (F-actin) or with transmission electron microscopy (TEM) (Fig. 1A–D). Interestingly, we also see Rab11 endosomes localising apically in follicle cells around the time of microvilli biogenesis (Fig. 1E,F). Given that Rab11 endosomes are known to be involved in endocytic recycling to the apical membrane, as well as in trans-Golgi to plasma membrane exocytic delivery (Jing and Prekeris, 2009; Rodriguez-Boulan and Macara, 2014) and microvillus formation in enterocytes (Knowles et al., 2015), we examined their role in trafficking Cad99C to the apical membrane by inducing Rab11 RNA interference (RNAi) in follicle cells. Knockdown of Rab11 resulted in loss of Cad99C from the apical membrane, suggesting that trafficking of Cad99C occurs through Rab11 endosomal transport (Fig. 1G). To rule out an indirect effect of Rab11 on Cad99C trafficking through misregulation of epithelial polarity, we tested the effect of Rab11 RNAi on markers of cell polarity. We found that the localisation of atypical protein kinase C (aPKC) and Dlg is not affected upon Rab11 knockdown (Fig. S1A). These results show that epithelial polarity is retained in Rab11 RNAi cells.

Rab11 endosomes use an array of adaptor proteins to bind to different motors for intracellular transport (Horgan and McCaffrey, 2009; Junutula et al., 2004; Meyers and Prekeris, 2002; Prekeris, 2003). Nuf has previously been shown to interact with Rab11 and to be required for its correct localisation to the cleavage furrow during cytokinesis (Cao et al., 2008; Riggs et al., 2003). Nuf is also known to directly interact with the minus-end motor Dynein to transport cargo towards microtubule minus-ends (Riggs et al., 2007). We found that Nuf localises apically in follicle cells (Fig. 1H). We therefore tested the requirement for Nuf and Dynein to transport Rab11 endosomes. *nuf* mutants and knockdown of Dynein both revealed mislocalisation of Rab11 endosomes from the apical membrane to the cytoplasm (Fig. 1I). These results demonstrate the importance of Nuf and Dynein for correct apical localisation of Rab11 endosomes in the follicle cell epithelium. We found that cell polarity is not affected in *nuf* mutants or Dynein RNAi follicle cells, as aPKC and Dlg are localised normally in both conditions (Fig. S1A,B).

**Fig. 2.**
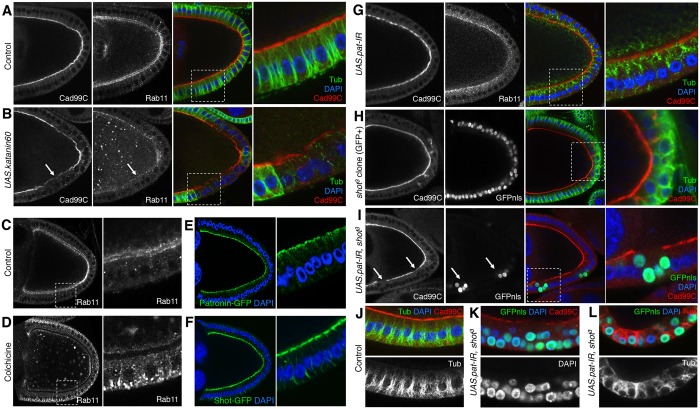
**Cad99C is transported apically along microtubules that are polarised by Patronin and Shot.** (A) Control egg chamber stained for Cad99C, Rab11, DAPI and Tubulin to show polarised microtubules. (B) Overexpression of Katanin60 causes microtubules to depolymerise, resulting in loss of Rab11 and Cad99C polarisation (arrows). Control egg chambers (C) or egg chambers treated with colchicine (D) to depolymerise microtubules; Rab11 polarisation is lost upon colchicine treatment. Expression of Patronin–GFP (E) and Shot–GFP (F) shows both proteins localise apically. Expression of Patronin RNAi (G) or mutation of *shot* (GFP-positive clone) (H) causes depolarisation microtubules, affecting Rab11 localisation and Cad99C protein levels. (I–L) Combined perturbation of Patronin and Shot (GFP-positive clone) results in loss of Cad99C from the apical membrane and causes severe defects in microtubule polarisation. Loss of polarised microtubules results in the mislocalisation of nuclei in these mutants, which gives the impression of multilayering of the follicle cells (arrows in I).

We next studied the requirement for microtubules in Rab11 endosome trafficking and microvilli biogenesis. We induced overexpression of the microtubule-severing protein Katanin 60 to trigger depolymerisation of most microtubules (Diaz-Valencia et al., 2011), and found that loss of microtubules led to accumulation of Rab11 endosomes in the cytoplasm and failure of Cad99C delivery (Fig. 2A,B). We also depolymerised the microtubules in follicle cells by treating the egg chambers with colchicine for 1 h. Control egg chambers had apical Rab11 localisation, whereas egg chambers treated with colchicine had endosomes accumulating basally in the follicle cells (Fig. 2C,D). Thus, the polarisation of Rab11 endosomes for delivery of Cad99C is a microtubule-dependent process.

To explore how the microtubules become polarised in follicle cells, we considered the roles of two microtubule-binding proteins Patronin and Shot. Patronin has been reported to bind minus-ends of microtubules through its C-terminal CKK domain and protect them from Kinesin-13-mediated degradation (Baines et al., 2009; Goodwin and Vale, 2010; Hendershott and Vale, 2014). Furthermore, in mammalian cells CAMSAP3 and CAMSAP2 have been shown to cooperate to organise epithelial-specific organisation of acentrosomal microtubules (Tanaka et al., 2012; Toya et al., 2016). Shot is a spectraplakin cytoskeletal protein, known to crosslink microtubules to the actin cytoskeleton (Applewhite et al., 2010; Lee and Kolodziej, 2002). Shot can bind F-actin through its N-terminal actin-binding domain and to microtubules through its C-terminal GAS2 domain (Applewhite et al., 2010; Lee and Kolodziej, 2002; Lee et al., 2000; Sun et al., 2001). We found that GFP-tagged Patronin and Shot localised apically in follicle cells, suggesting a potential role in polarising the microtubule cytoskeleton along the apical-basal axis of epithelial cells (Fig. 2E,F).

We depleted Patronin in follicle cells by RNAi, which produced a moderately disordered microtubule cytoskeleton, mildly affecting Rab11 trafficking (Fig. 2G). Cad99C localisation remained largely unaffected, likely due to a slow turnover rate of the protein (Fig. 2G). We next investigated the requirement of Shot by analysing mutants with the null allele *shot^3^*, which has previously been reported to cause occasional double layering in the follicle cell epithelium (Gregory and Brown, 1998; Röper and Brown, 2003). *shot^3^* moderately affected microtubule polarisation and Rab11 endosome trafficking, with a minimal effect on Cad99C localisation (Fig. 2H). Owing to the weak phenotypes of losing Patronin and Shot individually, we combined the two manipulations to see whether this caused a stronger phenotype. We found that perturbing both proteins severely affected microtubule organisation and led to loss of Cad99C from the apical membrane (Fig. 2I–L). We found that severe disruption of microtubule organisation often led to mis-positioning of nuclei in follicle cells, giving cells the appearance of multilayering, when they are actually still a monolayer. Our results indicate that Patronin and Shot work in parallel to polarise microtubules, and that microtubule polarisation is essential for apical delivery of Cad99C in follicle cells.

**Fig. 3.**
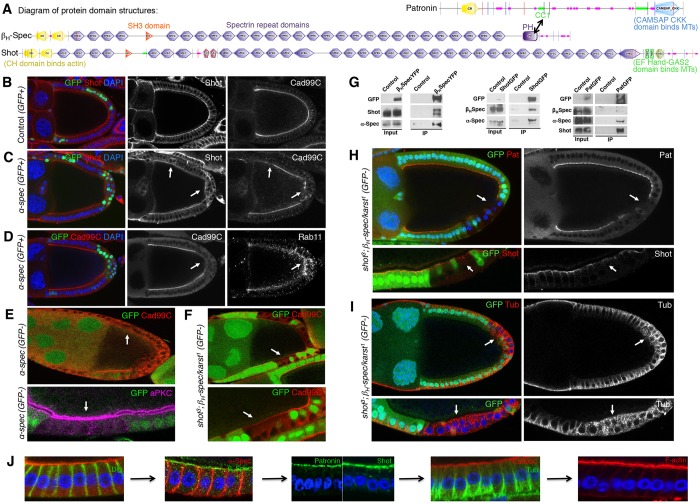
**The spectrin cytoskeleton is required to polarise Patronin and Shot in response to apical-basal polarity determinants.** (A) Schematic diagram of protein domain structures of Patronin, β_H_-Spectrin (Karst) and Shot. (B) Control egg chamber stained for Shot and Cad99C. Mutation of *α-spectrin* (GFP-positive clone) causes mislocalisation of Shot, Cad99C (C) and Rab11 (D) (arrows). (E) Top panel: *α-spectrin* mutants (GFP-negative clone) exhibit loss of Cad99C from the apical membrane (arrow). Bottom panel: *α-spectrin* mutants (GFP-negative clone) have normal aPKC polarisation but show loss of perivitelline space between the follicle cell membrane and the oocyte membrane (arrow), suggesting microvilli defects. (F) Double mutants for Shot and β_H_-Spectrin (GFP-negative clone) show loss of Cad99C from the apical membrane (arrows). (G) Left panel: co-immunoprecipitation (IP) of endogenous β_H_-Spectrin–YFP (β_H_SpecYFP) knock-in embryos with Shot and α-Spectrin. Middle panel: co-immunoprecipitation of UAS.Shot–GFP (ShotGFP) embryos with β_H_-Spectrin and α-Spectrin. Right panel: co-immunoprecipitation of Patronin–GFP (PatGFP) embryos with β_H_-Spectrin, α-Spectrin and Shot. (H) Top panel: double mutants for Shot and β_H_-Spectrin (GFP-negative clone) exhibit loss of Patronin from the apical domain (arrow). Bottom panel: Shot staining in Shot and β_H_-Spectrin double mutants (arrow). (I) Double mutants for Shot and β_H_-Spectrin (GFP-negative clone) show severe defects in microtubule polarisation (arrow). (J) Stepwise representation of events leading to the polarisation of Cad99C at the apical membrane for biogenesis of microvilli.

We next investigated the mechanism by which Patronin and Shot become polarised to the apical domain. We considered the role of spectrins in polarising Patronin and Shot to the apical membrane. The spectrin cytoskeleton is polarised in epithelial cells with α_2_β_H2_ heterotetramers localising to the apical domain and α_2_β_2_ heterotetramers localising to the basolateral domain (Thomas and Kiehart, 1994; Thomas and Williams, 1999; Zarnescu and Thomas, 1999). Several lines of evidence suggest that apical spectrins interact with Patronin and Shot. Firstly, a conserved region in mammalian CAMSAP1, known as the CC1 region, has been shown to bind the linker region adjacent to the PH domain of the long C-terminal variant of βII-spectrin *in vitro* (Fig. 3A) (King et al., 2014). Secondly, we identified α- and β_H_-Spectrin (also known as Karst) in the mass spectrometry analysis of Patronin or Shot-associated proteins in *Drosophila* (data not shown). Finally, Shot contains multiple spectrin repeat domains, suggesting that it might directly bind to spectrins (Fig. 3A) (Leung et al., 1999; Röper and Brown, 2003; Sun et al., 2001).

To test the requirement for the spectrin cytoskeleton in localising Shot and Patronin, we induced mutant clones for *α-spectrin* in the follicle cell epithelium. We found that loss of α-Spectrin caused mislocalisation of Shot from the apical domain, and also affected the localisation of Rab11 and Cad99C (Fig. 3B–E). Our observation is not an indirect affect of loss of polarity, as aPKC was not affected in *α-spectrin* mutants (Fig. 3E; Fig. S2A). Although the apical polarity was maintained, we found that *α-spectrin* mutant cells appeared to lose their perivitelline space and associate closely with the oocyte membrane, indicating that these cells might have defective microvilli (Fig. 3E, bottom panel). Consistent with this finding, we showed that loss of α-Spectrin prevented apical F-actin microvilli formation, but did not affect cortical F-actin in follicle cells (Fig. S2B,C). We confirm that loss of α-Spectrin or β-Spectrin could also cause a reduction in cell height, as recently reported (Fig. S2D–F; Ng et al., 2016).

We found that mutation of *β_H_-spectrin* or *shot* alone did not have a strong affect on Cad99C localisation (Fig. S2G,H). Owing to the similar structure and role of β_H_-spectrin and Shot in binding microtubules, we anticipated that there might be redundancy between the two proteins. To test this possibility, we analysed Cad99C localisation in double mutants of *shot* and *β_H_-spectrin.* Indeed, we found that Cad99C was lost from the apical membrane in the double mutants (Fig. 3F; Fig. S2I).

We next tested for interactions of spectrins with Patronin and Shot by performing co-immunoprecipitation experiments from *Drosophila* embryos expressing endogenously YFP-tagged β_H_-Spectrin. We found that tagged β_H_-Spectrin interacted strongly with two isoforms of Shot (Fig. 3G). Pulling down tagged β_H_-Spectrin also co-immunoprecipitated endogenous α-Spectrin. We also performed co-immunoprecipitation experiments in embryos expressing Shot–GFP and Patronin–GFP. We found that both Shot and Patronin bound to β_H_-Spectrin and α-Spectrin (Fig. 3G). Furthermore, we found that Patronin could bind to Shot (Fig. 3G). These results indicate that apical spectrins bind to Patronin and Shot, and act to recruit the two proteins to the apical membrane. Consistent with the data from the co-immunoprecipitation experiments, we showed that double mutants of *shot* and *β_H_-spectrin* lost polarisation of Patronin from their apical domains (Fig. 3H). In addition, these double mutants displayed severe defects in microtubule organisation (Fig. 3I), further supporting the notion that Shot and β_H_-Spectrin act redundantly to polarise microtubules in the follicle cell epithelium.

In epithelial cells, fundamental determinants of apical-basal cell polarity are responsible for polarising all other proteins in the cell. We sought to determine whether two key apical and basal polarity determinants, Cdc42 and Lgl [also known as L(2)gl], were important to organise polarisation of apical spectrins to direct the polarisation of downstream trafficking machinery for Cad99C. We found that mutants of *cdc42* and *lgl* exhibited mislocalisation of apical spectrins, Patronin and Shot, and also exhibited loss of Cad99C from the apical membrane (Fig. S3A–I). The loss of Cdc42 or Lgl caused a dramatic disruption of the epithelial tissue, making it difficult to determine whether these determinants act directly or indirectly to polarise spectrins, Patronin and Shot. Nevertheless, these findings suggest that apical-basal polarity determinants act upstream of Spectrin polarisation to control Patronin and Shot localisation and microtubule polarisation, which then directs apical trafficking of Cad99C for microvilli biogenesis (Fig. 3J).

**Fig. 4.**
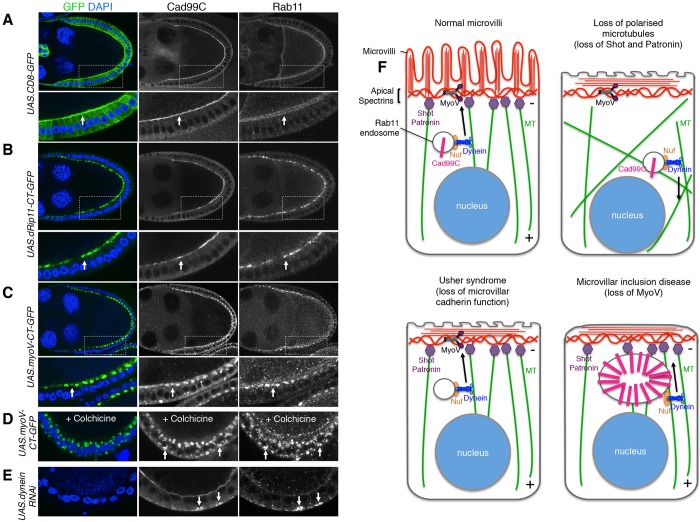
**The microvilli inclusion disease protein MyoV is required for apical delivery of Rab11 endosomes.** (A) Control egg chamber expressing *UAS.CD8GFP* and stained for Cad99C and Rab11. The arrows shows normal localisation in the control. Expression of dominant-negative Rip11–CT-GFP (B) or MyoV–CT-GFP (C) causes accumulation of Rab11 endosomes and Cad99C (arrows) near the apical region. (D) Colchicine treatment of egg chambers expressing dominant-negative MyoV–CT-GFP causes basal accumulation of Rab11 endosomes and Cad99C in follicle cells (arrows). (E) Basal accumulation of Rab11 endosomes and Cad99C also occurs in follicle cells expressing Dynein RNAi. (F) Model for normal trafficking and delivery of Cad99C to promote apical microvilli biogenesis. Defects in trafficking or delivery of Cad99C results in loss of Cad99C function and leads to diseases such as Usher syndrome Type 1 and microvillus inclusion disease.

Once Rab11 endosomes are transported apically along microtubules by the Nuf–Dynein motor complex, they must traverse the apical F-actin cortex to be delivered to the plasma membrane. We found that a different motor complex is required to transport the endosomes beyond the microtubule network. Myosin V (MyoV; Myo5a and Myo5b in humans) is a known actin-based motor that has been implicated in polarised membrane transport of Rab11 endosomes in both mammals and flies (Lapierre et al., 2001; Li et al., 2007). Additionally, the *Drosophila* Rab11-interacting protein (Rip11, known as Rab11FIP1 in humans) has also been shown to bind Rab11 endosomes, as well as interact in a complex with MyoV during Rhodopsin transport in developing photoreceptors in *Drosophila* (Li et al., 2007; Prekeris et al., 2000). Based on these interactions, we investigated the roles of MyoV and Rip11 in the apical delivery of Rab11 endosomes using dominant-negative lines of both proteins, Rip11–CT-GFP and MyoV–CT-GFP, which express a C-terminal GFP-tagged version of the proteins (Li et al., 2007). Expression of Rip11–CT-GFP and MyoV–CT-GFP caused accumulation of Rab11 and Cad99C in the sub-apical region of follicle cells, with MyoV causing a more severe effect (Fig. 4A–C). We found that Rab11 colocalised with Cad99C in these accumulated endosomes (Fig. 4B,C). We showed that disrupting the microtubules with colchicine in follicle cells expressing MyoV–CT-GFP caused the accumulated endosomes to redistribute basally (Fig. 4D), which is reminiscent of the basal endosomes found in Dynein RNAi cells (Fig. 4E), where Rab11 and Cad99C also colocalise. These results suggest that Rip11 and MyoV are dispensable for apical transport of Rab11 endosomes along microtubules but are required for their apical delivery through the F-actin cortex to the plasma membrane (Fig. 4F).

## DISCUSSION

Our results reveal a mechanism linking determinants of cell polarity with stepwise polarisation of the spectrin cytoskeleton, microtubule cytoskeleton and biogenesis of actin microvilli through apical trafficking of Cad99C. The results suggest that polarisation of the apical spectrin β_H_-Spectrin is dependent on polarity determinants, likely through interactions with the FERM domain proteins and the apical polarity determinant Crb (Fletcher et al., 2015; Médina et al., 2002). The spectraplakin Shot is highly similar to β_H_-Spectrin, and is able to bind to and colocalise with it at the apical domain of epithelial cells, suggesting that the two proteins might have a similar function. β_H_-Spectrin is linked to microtubules through Patronin, whereas Shot can directly bind microtubules. Consequently, redundancy is anticipated between β_H_-Spectrin and Shot, or between Patronin and Shot. Accordingly, we found that mutation of *β_H_-spectrin* only had a mild phenotype, whereas mutation of *α-spectrin* simultaneously disrupted both pairs of proteins in parallel and caused a drastic phenotype, completely disrupting the apical trafficking of Cad99C and microvillar biogenesis. More importantly, double mutants for *shot* and *β_H_-spectrin* had a more severe effect on microtubule and Cad99C localisation than either alone, therefore demonstrating that the two proteins act in a redundant fashion.

Downstream of the spectrin cytoskeleton, Patronin and Shot are required in parallel to drive apical-basal polarisation of microtubules, which is then responsible for orienting the apical transport of Cad99C, within Rab11 endosomes, by the Dynein motor protein. Eliminating microtubules from cells by overexpressing Katanin60 results in loss of Nuf–Dynein-based apical Rab11 endosome transport and failure to efficiently deliver Cad99C to the apical membrane. The effect on Cad99C polarisation is not an indirect effect of loss of polarity due to impaired Rab11 and Dynein function in localising the apical polarity determinant Crumbs to the apical membrane (Horne-Badovinac and Bilder, 2008; Li et al., 2008) because, firstly, polarity is maintained in cells expressing Rab11 or Dynein RNAi, as indicated by the normal localisation of aPKC and, secondly, loss of Crb does not strongly affect cell polarity in the follicle cell epithelium owing to redundancy with Bazooka (Fletcher et al., 2012). Our results indicate that even under conditions with severe depletion of microtubules, the overall shape of the follicle cell epithelium is relatively normal, indicating that polarised microtubules are required to influence formation of apical microvilli, rather than for other functions of the actin cytoskeleton in epithelial cells. Similarly, we do not see strong effects on cell shape upon loss of either Patronin or Shot (or both), raising questions over the claimed requirement for Patronin homologs and microtubules in formation or maintenance of adherens junctions epithelial cells in culture (Chen et al., 2003; Le Droguen et al., 2015; Meng et al., 2008; Stehbens et al., 2006).

The final step in delivery of Cad99C to the apical membrane also requires actin-based transport through the action of Rip11–MyoV complex. Compromising normal MyoV function in *Drosophila* follicle cells by expressing a dominant-negative version of the protein, results in loss of Rab11 polarisation from the apical membrane and its abnormal accumulation in the sub-apical region. This phenotype in *Drosophila* shows similarities with the human microvillus inclusion disease, where mutations in the Myo5b gene also cause loss of Rab11 endosomes from the apical membrane (Knowles et al., 2014; Lapierre et al., 2001).

In summary, our results reveal how the spectrin cytoskeleton acts to polarise microtubules in epithelial cells, and how polarised microtubules then direct trafficking of Rab11 endosomes carrying Cad99C to the apical membrane. This process relies on a hierarchy of events, and disruption at any stage can lead to failure in delivering Cad99C to the apical membrane, resulting in defective biogenesis of microvilli. Our findings are directly relevant to human diseases such as Usher's Syndrome Type 1 and microvillus inclusion disease, helping to outline the molecular and cellular basis for these conditions.

## MATERIALS AND METHODS

Mitotic clones in follicle cells were generated using the FLP-FRT site-specific recombination system and were either marked negatively (absence of GFP) or positively (presence of GFP) with the mosaic analysis with a repressible cell marker (MARCM) technique (Lee and Luo, 1999; Xu and Rubin, 1993). Newly eclosed females were heat-shocked once at 37°C for 1 h and ovaries were dissected 5 days after heat-shock.

The ‘Flip-out’ actin.FRT.CD2.FRT.Gal4/UAS system was used to express the UAS-Rab11IR construct. To express the transgenes, newly eclosed females were heat-shocked at 37°C for 10 min and ovaries were dissected 2 days after heat-shock. Expression of other *UAS*-driven transgenes in follicle cells was achieved with the follicle-cell-specific Gal4 drivers GR1.Gal4 and Traffic Jam.Gal4 (Tj.Gal4), as well as by using the MARCM system. *w* OR flies were used as the wild-type stock.

### Fly stocks

RNAi lines were ordered from the Vienna *Drosophila* Resource Center: Patronin IR (VDRC 27654) and Dynein IR (VDRC 28054). The Rab11 RNAi line was generated by Ruth Brain (our laboratory) in the laboratory. UAS.shot-GFP, FRT42B shot^3^, Ubi.patronin-GFP, UAS.katanin60, GR1.Gal4, FRT19A cdc42^3^ and FRT40A lgl^4^ were ordered from Bloomington *Drosophila* Stock Center. UAS.myoV-CT-GFP and UAS.dRip11-CT-GFP lines were gifts from Don Ready (Purdue University, USA) (Li et al., 2007). The Rab11–YFP line was a gift from Marko Brankatschk, MPI-CBG, Dresden, Germany. Kst–YFP (DGRC 115-285), Tj.Gal4 (DGRC 104-055) and FRT80B nuf (DGRC 111-536) were ordered from the *Drosophila* Genetic Resource Center, Kyoto. The following strains were used as in previous studies: α-spec^e226^ (Hüelsmeier et al., 2007), kstd1113 (Campos et al., 2010), kst1 (Thomas and Kiehart, 1994), Nod.lacZ and Kin.lacZ (Clark et al., 1997). A list of *Drosophila* genotypes used in each figure is presented in Table S1.

### Immunostaining of ovaries and microscopy

Ovaries were dissected in PBS, fixed for 20 min in 4% paraformaldehyde in PBS, washed for 30 min in PBS with 0.1% Triton X-100 (PBST) and blocked for 30 min in 5% normal goat serum in PBST (PBST with NGS). Primary antibodies were diluted in PBST with NGS and samples were incubated overnight at 4°C.

For Crumbs staining, ovaries were fixed for 10 min in 8% paraformaldehyde in PBS, washed in methanol for 5 min, washed for three time for 20 min each in PBST and for 5 min in 1% SDS, rinsed in PBS three times and blocked for 30 min in 5% PBST with NGS. The rest of the staining was carried out as described previously (Fletcher et al., 2012).

Primary antibodies used were: rabbit anti-aPKC, mouse anti-Dlg, mouse anti-α-Spectrin, rabbit anti-βH-Spectrin, rabbit anti-Cad99C, guinea pig anit-Cad99C, guinea pig anti-Shot, rabbit anti-Rab11, mouse anti-Crumbs, mouse anti-α-tubulin and rabbit anti-Nuf. Full details of the primary antibodies are available in Table S2. Phalloidin-TRITC (Sigma) was used to stain F-actin. Secondary antibodies (all from Molecular Probes, Invitrogen) were used at 1:500 for 2 h at room temperature along with DAPI staining at 1 µg/ml and then washed multiple times in PBST. Samples were mounted on slides in Vectashield (Vector labs). Images were acquired on a Zeiss LSM710 confocal microscope using 40× or 63× oil immersion objectives, and processed using Adobe Photoshop. Optical cross-sections through the middle of egg chambers are shown in all figures.

### Colchicine treatment

Wild-type egg chambers were cultured in imaging medium containing Schneider's medium (Invitrogen), Insulin (Sigma), heat-inactivated fetal calf serum (FCS; GE Healthcare), Trehalose (Sigma), adenosine deaminase (Roche), methoprene (Sigma) and ecdysone (Sigma) (Prasad et al., 2007), with 0.2 mg/ml of colchicine or ethanol (for control) for 1 h at room temperature. After treatment, samples were fixed and processed normally for imaging.

### Co-immunoprecipitation

For co-immunoprecipitation experiments, *Drosophila* Karst YFP knock-in embryos (DGRC 115285), Wiso embryos, and embryos expressing Patronin–GFP or Shot–GFP were collected over 24 h at 22°C before being lysed in buffer containing 10 mM Tris-HCl pH 7.5, 150 mM NaCl, 0.5% NP-40 and 0.5 mM EDTA (Chromotek), plus PhosSTOP Phosphatase Inhibitor Cocktail Tablets (Roche), protease inhibitor cocktail (Roche), 0.1 M NaF and 1 mM PMSF. Samples were left on ice to solubilise for 30 min, before being centrifuged at high speed (14,000 rpm in a desktop centrifuge for 30 min at 4°C). The supernatant was collected, pre-cleared and incubated with GFP Trap-M beads (Chromotek).

Western blots were probed with mouse anti-GFP, guinea pig anti-Shot, rabbit anti-Patronin, mouse anti-α-Spectrin and rabbit anti-βH-Spectrin antibodies (details in Table S3; see Fig. S4 for complete western blots and for siRNA knockdown experiments in human cells), before being detected with chemiluminescence (GE Healthcare).

### Electron microscopy of *Drosophila* egg chambers

*Drosophila* egg chambers were fixed in 2.5% glutaraldehyde and 4% formaldehyde in 0.1 M phosphate buffer (pH 7.4) and then processed for transmission electron microscopy (TEM) and serial block-face scanning electron microscopy (SBFSEM). Samples were prepared using the National Center for Microscopy and Imaging Research (NCMIR) method (Deerinck et al., 2010). For TEM, 70-nm sections were cut using a UCT ultramicrotome (Leica Microsystems) and collected on formvar-coated slot grids. No post-staining was required owing to the density of metal deposited using the NCMIR protocol. Images were acquired using a 120 kV Tecnai G2 Spirit Biotwin (FEI Company) and Orius CCD camera (Gatan Inc.).
