# Correction: Shot and Patronin polarise microtubules to direct membrane traffic and biogenesis of microvilli in epithelia

**DOI:** 10.1242/jcs.206912

**Published:** 2017-07-01

**Authors:** Ichha Khanal, Ahmed Elbediwy, Maria del Carmen Diaz de la Loza, Georgina C. Fletcher, Barry J. Thompson

There was an error published in *J. Cell Sci.* **129**, 2651-2659.

In the abstract of this paper, there was a typographical error in the protein name CAMSAP3. The correct sentence should read:

Core apical-basal polarity determinants polarise the spectrin cytoskeleton to recruit the microtubule-binding proteins Patronin (CAMSAP1, CAMSAP2 and CAMSAP3 in humans) and Shortstop [Shot; MACF1 and BPAG1 (also known as DST) in humans] to the apical membrane domain.

The online version of the article has been corrected accordingly.

We apologise to the authors and readers for any confusion that this error might have caused.

